# Prenatal selective serotonin reuptake inhibitor (SSRI) exposure induces working memory and social recognition deficits by disrupting inhibitory synaptic networks in male mice

**DOI:** 10.1186/s13041-019-0452-5

**Published:** 2019-04-01

**Authors:** Weonjin Yu, Yi-Chun Yen, Young-Hwan Lee, Shawn Tan, Yixin Xiao, Hidayat Lokman, Audrey Khoo Tze Ting, Hasini Ganegala, Taejoon Kwon, Won-Kyung Ho, H. Shawn Je

**Affiliations:** 1 Molecular Neurophysiology Laboratory, Signature Program in Neuroscience and Behavioral Disorders, Duke-National University of Singapore (NUS) Medical School, 8 College Road, Singapore, 169857 Singapore; 2 Department of Physiology, Seoul National University College of Medicine, Seoul, 03080 Republic of Korea; 3 Department of Physiology, Yong Loo Lin School of Medicine, National University of Singapore, Singapore, 117597 Singapore; 4 Department of Biomedical Engineering, School of Life Science, Ulsan National Institute of Science and Technology (UNIST), UNIST-gil 50, Ulsan, 44919 Republic of Korea

**Keywords:** Prenatal, Serotonin (5-HT), Selective serotonin reuptake inhibitor (SSRI), Fluoxetine, Working memory, Social recognition, Serotonin 2A receptor (5-HT_2A_R)

## Abstract

Selective serotonin reuptake inhibitors (SSRIs) are commonly prescribed antidepressant drugs in pregnant women. Infants born following prenatal exposure to SSRIs have a higher risk for behavioral abnormalities, however, the underlying mechanisms remains unknown. Therefore, we examined the effects of prenatal fluoxetine, the most commonly prescribed SSRI, in mice. Intriguingly, chronic in utero fluoxetine treatment impaired working memory and social novelty recognition in adult males. In the medial prefrontal cortex (mPFC), a key region regulating these behaviors, we found augmented spontaneous inhibitory synaptic transmission onto the layer 5 pyramidal neurons. Fast-spiking interneurons in mPFC exhibited enhanced intrinsic excitability and serotonin-induced excitability due to upregulated serotonin (5-HT) 2A receptor (5-HT_2A_R) signaling. More importantly, the behavioral deficits in prenatal fluoxetine treated mice were reversed by the application of a 5-HT_2A_R antagonist. Taken together, our findings suggest that alterations in inhibitory neuronal modulation are responsible for the behavioral alterations following prenatal exposure to SSRIs.

**Electronic supplementary material:**

The online version of this article (10.1186/s13041-019-0452-5) contains supplementary material, which is available to authorized users.

## Introduction

Anti-depressants are commonly prescribed to treat major depression and post-traumatic stress disorder. Currently, 17% of pregnant women experience major depression, and approximately 10% of these women use anti-depressants [1–3]. The most commonly prescribed anti-depressants, selective serotonin reuptake inhibitors (SSRIs), are believed to increase the ambient level of 5-hydrotryptamine (5-HT, serotonin) in synaptic clefts by preventing its reabsorption [4–6]. However, the exact mechanism by which SSRIs mitigates depression remains unknown. A recent systematic review showed that the potential adverse effects of SSRIs might outweigh their beneficial effects on depression [7–9]. In addition, fluoxetine (FLX), one of the most widely used SSRIs with a moderately long half-life (t _½_ = 48 h), can cross the placental and blood-brain barriers and is also detected in breast milk, suggesting potential accumulation of FLX as well as 5-HT in the fetal brain [10]. However, little is known about the safety of FLX use during pregnancy. Moreover, the long-term consequences of prenatal FLX exposure for adverse behavioral outcomes in offspring are uncertain and sometimes conflicting; these conflicting findings are likely due to the independent association between maternal depression and negative pregnancy outcomes in human [11, 12].

Ansorge et al. firstly observed that postnatal FLX exposure produced anxiety behaviors and disrupted learning in rodent offspring [13, 14]. In subsequent studies, manipulations of brain 5-HT levels during early development produced abnormal neuronal circuit formation in the cortex and promoted aggressive or anxiety-related behaviors [15–19]. However, a thorough assessment of animal behaviors induced by prenatal SSRI exposure has not been performed. Furthermore, the underlying molecular and circuit mechanisms of these behavioral changes have not been investigated and, for this reason, no rescue experiment has been performed in offspring exposed to SSRIs during prenatal period.

Using a combination of behavioral analyses and electrophysiological investigations of affected neuronal circuits, we examined how chronic prenatal exposure to exogenous FLX influences animal behaviors and neuronal circuitry and function. We observed impaired social recognition and working memory in male mice chronically exposed to FLX. Furthermore, we observed reduced frequencies in spontaneous excitatory postsynaptic currents recorded from layer (L) 5 pyramidal neurons in the prelimbic cortex of affected mice. Intriguingly, these reduced excitatory neuronal activities were caused by enhanced serotonergic modulation of fast-spiking (FS) interneurons in L5 due to enhanced 5-HT_2A_ receptors (5-HT_2A_Rs). Moreover, the acute treatment with the 5-HT_2A_R antagonist MDL100907 (MDL) normalized the impaired social recognition and working memory impairment in these animals [20].

## Results

### Prenatal fluoxetine treatment induced deficits in working memory and social recognition

**Fig. 1:**
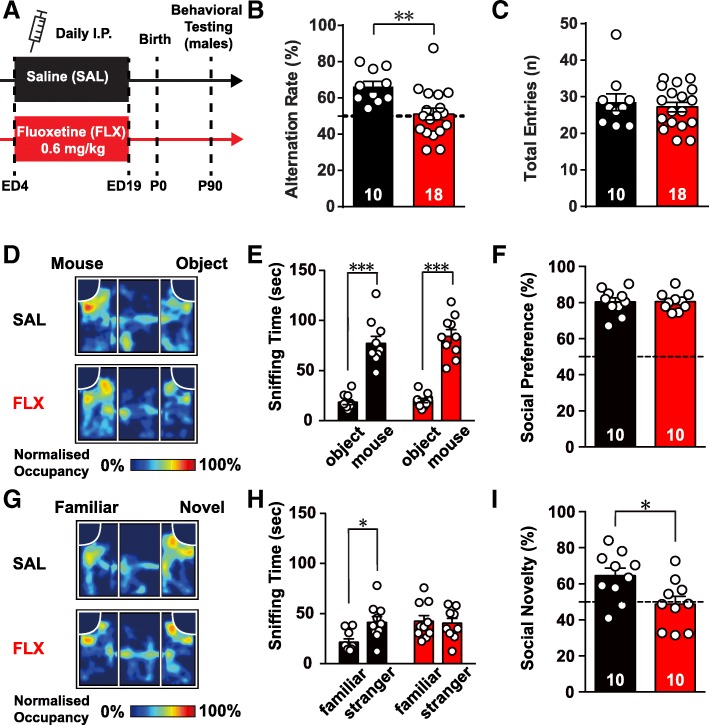
Prenatal exposure to fluoxetine induces deficits in executive functions in male offspring. (**a**) Schematic diagram of the experimental design. From embryonic day 4 (ED4) to ED19, pregnant females received daily injections of either fluoxetine (FLX, 0.6 mg/kg/day) or saline (SAL). Male offspring were subjected to behavioral testing at 8–12 weeks of age. (**b**) Bar plot of spontaneous alternation rate on the Y-maze. (**c**) Bar plot of the total number of entries into all arms of the Y-maze. (**d**) Representative heat map images of results of the three-chamber social interaction test with a novel mouse and object. The occupancy rate was normalized to the region with peak occupancy in the arena. (**e**) Bar plot of the time spent sniffing the novel mouse and object in the three-chamber social interaction task. (**f**) Bar plot of the social preference index (time spent sniffing mouse/total time spent sniffing the mouse and the object). The dotted line indicates an equal preference (50%) (**g**) Representative heat map images of the results of the three-chamber social interaction tests with a familiar mouse and novel mouse. (**h**) Bar plot of the times spent sniffing the familiar mouse and novel mouse in the three-chamber social interaction task. (**i**) Bar plot of the social novelty preference index (time spent sniffing the novel mouse/total time spent sniffing both mice). Data are presented as means ± SEM. (**e**) and (**h**) Two-way repeated measures ANOVA. (**b**), (**c**), (**f**) and (**i**) Unpaired t-test. * *p* < 0.05, ** *p* < 0.01, *** *p* < 0.001

We subjected pregnant mice to daily intraperitoneal (i.p.) injections of 0.6 mg/kg FLX or saline (SAL) from embryonic day (ED) 4 to ED19 to examine behavioral changes in mice exposed to SSRIs during the prenatal period (Fig. 1a) [21]. The mean number of pups born per litter, percentages of male pups per litter, and average body weights of mice at postnatal day 21 (P21) and P60 were not significantly different between FLX-treated litters and SAL-treated, control litters (Table 1). The FLX-treated mice exhibited normal spontaneous exploratory behavior in terms of the total distance traveled (Additional file 1: Figure S1A), but spent less time in the center zone (t_(26)_ =2.12, *p* < 0.001; Additional file 1: Figure S1B), indicating a potential sign of anxiety-like behaviors. We tested the mice using the elevated zero maze and light-dark box test to examine anxiety-like behaviors (Additional file 1: Figures S1C, D). However, FLX-treated mice did not exhibit differences in the time spent in the open arms of the elevated zero maze (Additional file 1: Figure S1C) or total transitions in the light-dark box (Additional file 1: Figure S1D), suggesting normal anxiety levels in FLX-treated mice.

**Table 1:** Effects of prenatal SAL and FLX treatment on number, gender composition and weight of offspring

	SAL	FLX
Total number of litters	22	23
Total number of offspring	104	96
Total number of male mice	58	49
Average number of pups per litter	4.73 ± 0.44	4.17 ± 0.37
Average number of male pups per litter	2.64 ± 0.33	2.13 ± 0.21
Percentage of male pups in each litter	55.88 ± 4.98%	54.26 ± 4.19%
Weight of male mice at P28	16.44 ± 0.41 g, *n* = 20	15.89 ± 0.33 g, *n* = 23

To further examine the role of prenatal FLX in working memory and cognitive function, we subjected FLX-treated mice to the Y-maze spontaneous alternation task [22]. Briefly, both control and FLX treated mice were allowed to freely access three arms of the Y-maze for 10 min. Mice prefer to explore a previously uncharted arm of the maze rather than returning to a previously visited arm [23]. Intriguingly, the alternation rate of prenatally FLX-treated mice was lower than that of SAL-treated mice (t [24] =3.05, *p* < 0.01; Fig. 1b) without changes in general activity, as measured by the total arm entries (*p* > 0.05; Fig. 1c). To test whether this reduced alternation rate was due to either behavioral perseverance or recognition of a new environment, we performed the novel object recognition test. Interestingly, both prenatally SAL- and FLX-treated mice were able to distinguish the novel and familiar objects, as assessed by the time spent exploring the novel object (Additional file 1: Figure S1E). Additionally, there was no significant difference in grooming behaviors between the two groups, indicating that the behavioral perseverance of FLX-treated mice was not associated with repetitive or obsessive-compulsive behaviors (Additional file 1: Figure S1F).

Next, we subjected the two groups of mice to the social interaction test by using a 3-chamber apparatus [25, 26]. During the 10-min habituation phase, neither groups showed any side preference in the 3-chamber apparatus as reflected by the lack of differences in the time spent sniffing two empty wire pencil holders in the left and right chambers (*p* > 0.05; Additional file 1: Figures S1G, H). Over the next 10 min, both groups spent significantly more time sniffing the juvenile male mouse (social stimulus) than the dummy object (*t* [9] =7.72, *p* < 0.001 for SAL; *t* [9] =10.39, *p* < 0.001 for FLX; Fig. 1d-f). During the last 10 min, prenatally SAL-treated mice spent significantly more time sniffing the novel juvenile male mouse than the familiar juvenile mouse (*t* [9] =3.28, *p* < 0.01), confirming a preference for social novelty in SAL mice. In contrast, prenatally FLX-treated mice failed to show this preference, as indicated by the similar time spent in investigating the novel and familiar juvenile mice (*t* [9] = 0.25, *p* = 0.81; Fig. 1g-i). Taken together, these data indicate that prenatally FLX-treated mice exhibited deficits in working memory and social novelty recognition.

### Increased frequency of spontaneous and miniature inhibitory synaptic currents in layer 5 pyramidal neurons in the prefrontal cortex of FLX-treated mice

**Fig. 2:**
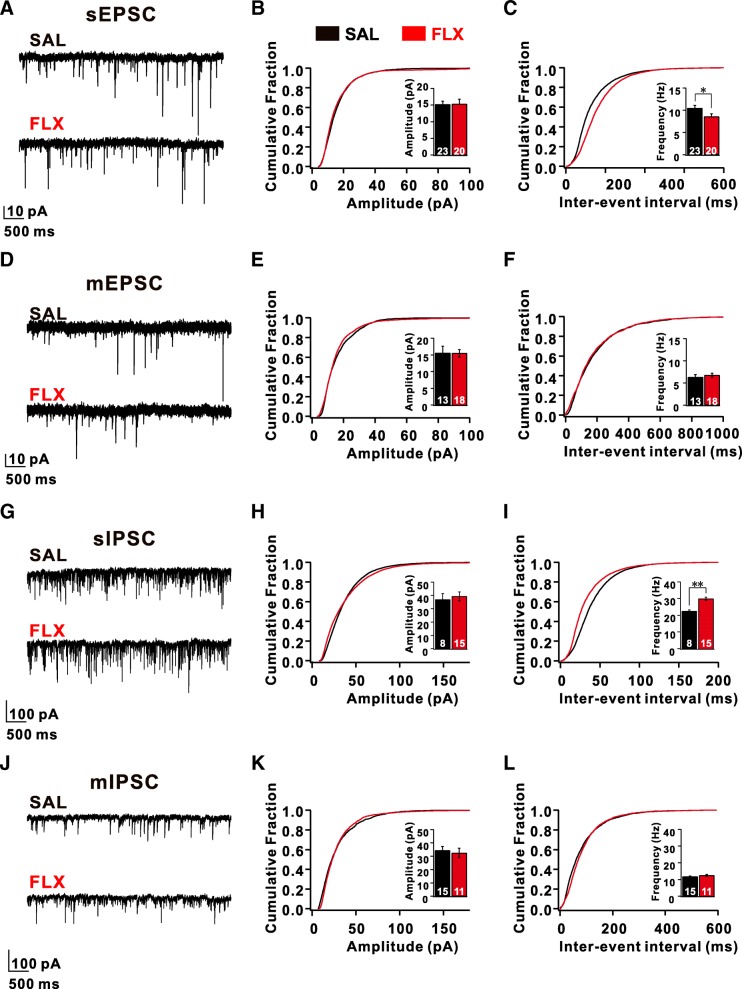
Fluoxetine induces an increase in the number of inhibitory inputs to excitatory neurons and reduced excitatory basal transmission in the L5 pyramidal neurons of the prelimbic cortex of FLX-treated mice. (**a**) Representative current traces depicting spontaneous excitatory post-synaptic currents (sEPSCs) obtained from L5 pyramidal neurons in the prelimbic cortex (PrL) pyramidal neurons of SAL- and FLX-treated (red) mice. (**b** and **c**) Plots of the cumulative distributions of sEPSC (**b**) amplitudes and (**c**) inter-event intervals obtained from SAL- (black) and FLX-treated (red) mice. (**d**) Representative current traces depicting mEPSCs obtained from SAL- and FLX-treated mice. (**e** and **f**) Plots of the cumulative distribution plots of mEPSC (**e**) amplitudes and (**f**) inter-event intervals. (**g**) Representative current traces depicting sIPSCs obtained from SAL- and FLX-treated mice. (**h** and **i**) Plots of the cumulative distribution plots of sIPSC (**h**) amplitudes and (**i**) inter-event intervals. (**j**) Representative current traces depicting mIPSCs obtained from SAL- and FLX-treated mice. (**k** and **l**) Plots of the cumulative distribution of mIPSC (**k**) amplitudes and (**l**) inter-event intervals. Data are presented as mean ± SEM. All data were analyzed using the Mann-Whitney U test. *p < 0.05, **p < 0.01

Although the circuit mechanism underlying social behavior phenotypes observed in prenatally FLX-treated mice is not obvious [27, 28], the deficits in working memory observed in the Y-maze spontaneous alternation task prompted us to characterize neuronal activities in the prefrontal cortex (PFC), which is functionally analogous to the dorsolateral PFC, a critical area known for working memory in humans [29]. Within the PFC, information is transmitted top-down via pathways from L2/3 pyramidal neurons to pyramidal neurons in L5 [30]. We first characterized the morphology of L5 pyramidal neurons, which send the major corticofugal outputs from the PFC network. We did not observe any differences in layer formation, the number of neurons, dendritic complexity, and the number of dendritic spines in L5 pyramidal neurons between FLX-treated and SAL-treated mice (Additional file 1: Figure S2). Next, using whole-cell patch-clamp recordings, we characterized the intrinsic properties of L5 pyramidal neurons within the prelimbic area (PrL), which is homologous to the dorsolateral PFC in primates [30]. We first measured spontaneous excitatory postsynaptic currents (sEPSCs) in L5 pyramidal neurons (Fig. 2a-c). The frequency of sEPSCs recorded from FLX-treated mice was significantly decreased by 18% compared with those recorded from SAL-treated mice, whereas the amplitude of sEPSCs was unaltered (SAL: 10.46 ± 0.50 Hz, 15.23 ± 0.92 pA; FLX: 8.56 ± 0.51 Hz, 15.32 ± 1.45 pA) (Fig. 2a-c). This indicates that spontaneous excitatory synaptic transmission in the PFC was decreased in FLX-treated mice. To further explore the mechanism of changes in sEPSCs in FLX-treated mice, we recorded miniature excitatory postsynaptic currents (mEPSCs) and the neuronal excitability of L5 pyramidal neurons. Intriguingly, the frequency and amplitude of mEPSCs recorded from FLX-treated mice were not significantly different from mEPSCs recorded from SAL-treated mice (SAL: 6.34 ± 0.60 Hz, 15.61 ± 2.01 pA; FLX: 6.80 ± 0.34 Hz, 15.53 ± 1.11 pA) (Fig. 2d-f). More strikingly, most parameters of intrinsic neuronal properties (input resistance, resting membrane potentials, afterhyperpolarization amplitude, and the threshold of action potentials (APs)) of the L5 neurons of FLX-treated mice were not significantly altered (Additional file 1: Figure S3). These data indicate that the decreased spontaneous excitatory network activities did not arise from intrinsic changes in excitatory neurons in the prefrontal cortex of FLX-treated mice.

To test whether inhibitory synaptic transmission in the PFC was affected in FLX-treated mice, we recorded both spontaneous and miniature inhibitory postsynaptic currents (sIPSCs and mIPSCs, respectively) in L5 pyramidal neurons within the PrL [30]. Surprisingly, we observed a significant increase in the frequency of sIPSCs (SAL: 22.46 ± 0.72 Hz; FLX: 29.99 ± 0.96 Hz, *p* < 0.01), but not the amplitude (SAL: 36.88 ± 4.57 pA; FLX: 39.29 ± 3.36 pA) (Fig. 2g-i). In contrast, neither the frequency nor the amplitude of mIPSCs were affected in L5 neurons from FLX-treated mice (Fig. 2j-l). These data indicate that prenatal FLX exposure increased spontaneous inhibitory network activity in L5 pyramidal neurons within the PrL.

### Increased excitability and serotonergic modulation of L5 fast-spiking interneurons in PrL of FLX-treated mic

The mPFC receives dense serotonergic innervation from the raphe nuclei, and both pyramidal and interneurons within the mPFC express several 5-HT receptor subtypes, with a particularly high density of 5-HT1_A_ and 5-HT2_A_Rs [31–33]. Chronic increases in ambient synaptic 5-HT due to SSRI-mediated blockade of the serotonin transporter (5-HTT) could potentially result in desensitization, internalization, or compensatory level changes via transcription and translations of certain 5-HT receptors [24, 34]. To test this hypothesis, qRT-PCR was performed on the PrL tissues from either SAL-treated or FLX-treated dams. The qRT-PCR results showed a significant increase in the levels of the 5-HT_2A_R mRNAs (SAL vs FLX fold change in 5-HT_2A_R: 1.49 ± 0.14; *p* = 0.044, unpaired t-test; SAL, *n* = 5; FLX, *n* = 5). Although not statistically significant, there was an upward trend in the level of the 5-HT_1A_R mRNAs (SAL vs FLX fold change in 5-HT_1A_R: 1.48 ± 0.17; *p* = 0.054, unpaired t-test; SAL, *n* = 5; FLX, *n* = 5)(Additional file 1: Figures S4A, B). In contrast, we did not observe any significant changes in the mRNA expression levels of other 5-HT receptors and transporters in both qRT-PCR and microarray analyses (Additional file 1: Figures S4C, D).

We wondered whether the upregulation of 5-HT _2A_Rs resulted in increased spontaneous inhibitory synaptic transmission in L5 excitatory pyramidal neurons. To address this question, we first investigated functional changes in sIPSCs recorded from L5 pyramidal neurons upon acute exogenous 5-HT treatment. Consistent with results from previous studies [35, 36], treatment with exogenous 5-HT (30 μM) significantly enhanced both the frequency and amplitude of sIPSCs in L5 pyramidal neurons in SAL-treated mice by 32.74 ± 6.65% and 29.22 ± 11.09%, respectively (n = 5, *p* < 0.05, Additional file 1: Figures S4E, F). Intriguingly, L5 pyramidal neurons in FLX-treated mice exhibited substantial increases in the frequency and amplitude of sIPSCs (53.25% ± 7.85 and 36.93 ± 6.22%, *n* = 5, *p* < 0.05, Additional file 1, Figures S4E, F).

**Fig. 3:**
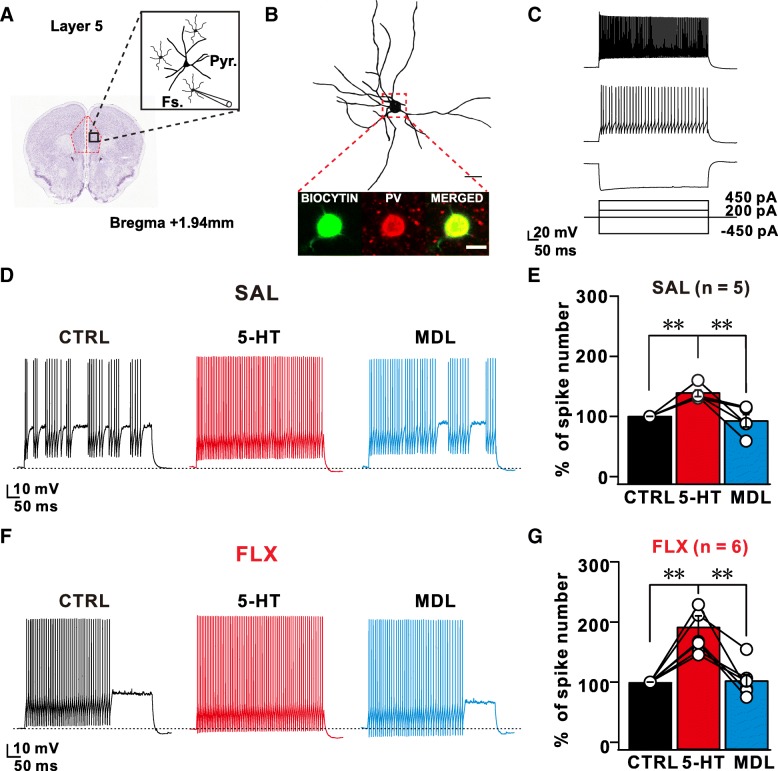
Increased excitability and serotonergic modulation of FS interneurons in PrL of FLX-treated mice. (**a**) Schematic diagram of the mouse PFC as outlined by the red dashed lines. The black box indicates a high magnification view of the neurons that were patched. We performed whole-cell patch-clamp recordings of putative fast-spiking (FS) interneurons in L5 of the PrL. (**b**) Representative image of a biocytin-filled FS interneuron in L5. Inset – co-staining for biocytin and parvalbumin, a marker of FS interneurons. (**c**) Characteristic responses of FS interneurons from SAL-treated mice to current injections (− 450 pA, 200 pA, and 450 pA) showing low adaptation to repeated firing. (**d**) Representative traces of FS interneurons from SAL-treated mice showing responses to current injections (200 pA) at baseline (CTRL), during the bath application of 5-HT (5-HT), and the bath application of the 5-HT_2A_R antagonist MDL. (**e**) Bar plot summarizing the effects of the 5-HT and MDL treatment on FS interneurons in SAL-treated mice (**f**) Representative traces of FS interneurons from FLX-treated mice show the responses to current injections (200 pA) under CTRL, 5-HT and MDL conditions. (**g**) Bar plot summarizing the effects of the 5-HT and MDL treatments on FS interneurons in FLX-treated mice. Data are presented as means ± SEM. All data were analyzed using the Wilcoxon signed ranks test. **p < 0.01

Next, we recorded the intrinsic excitability and firing properties of FS inhibitory neurons before and after 5-HT application to further investigate the effects of 5-HT on L5 FS inhibitory neurons that are critical for shaping cortical circuit activity by projecting their inhibitory outputs onto the L5 pyramidal neurons within the PFC [37–39](Fig. 3a). As shown by immunohistochemical staining, the recorded FS interneurons were positive for parvalbumin (PV) (Fig. 3b) and exhibited a characteristic 224-Hz firing at a 450pA current injection (Fig. 3c). Next, we applied a series of incremental square depolarizing pulses to L5 FS neurons from SAL or FLX mice before and during 5-HT application (Fig. 3c-g). 5-HT application significantly increased the spike frequency of L5 FS neurons at each injected current step in both SAL and FLX mice, and this increase in spike frequency was normalized after washout. Intriguingly, compared with L5 FS neurons from the SAL group, L5 FS neurons from the FLX group showed a larger increase in spike frequency in response to the 5-HT treatment (SAL: 38.57 ± 5.4% vs FLX: 92.05 ± 17.99%) (Fig. 3d-g). This increased frequency observed in L5 FS neurons was abolished by a subsequent treatment with MDL, a specific antagonist of 5-HT_2A_Rs (1 μM), indicating that the increased responsiveness of 5-HT_2A_R in L5 FS neurons resulted in a 5-HT-dependent increase in AP frequency (Fig. 3d-g). In contrast, co-treatment with 5-HT_1A_R antagonists (WAY-100135, 10 μM) and 5-HT did not affect 5-HT-mediated changes in the spike frequency of L5 FS interneurons (Additional file 1: Figures S5A-D). Thus, 5-HT-mediated changes in acute spike frequency were modulated by 5-HT_2A_Rs in the L5 FS interneurons of FLX-treated mice and subsequently increased sIPSCs in L5 pyramidal neurons.

We further tested the effect of 5-HT treatment on L5 pyramidal neurons. Compared with FS interneurons, L5 pyramidal neurons showed a significantly reduced spike frequency in response to 5-HT application (Additional file 1: Figures S6A-C). Furthermore, no significant difference in the 5-HT-mediated reduction in the sEPSC frequency in L5 pyramidal neurons was observed between SAL- and FLX-treated mice (Additional file 1: Figures S6D, G).

### Behavioral deficits of FLX-treated mice were ameliorated by acute treatment with 5-HT_2A_R antagonists

**Fig. 4:**
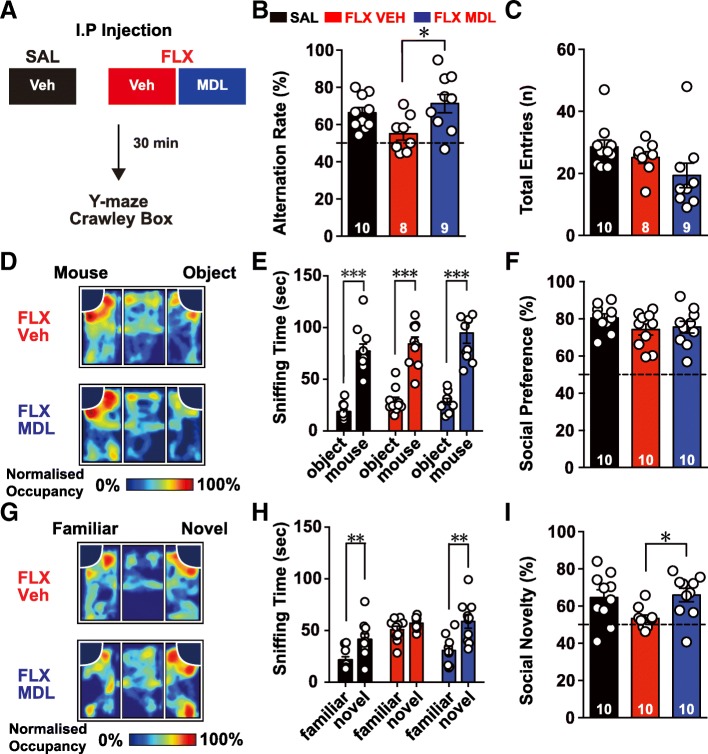
Prenatal FLX-induced deficits in executive function are rescued by a 5-HT_2A_R antagonist. (**a**) Schematic diagram of the drug treatment procedure. Prenatally SAL-treated mice were injected with vehicle (VEH), whereas prenatally fluoxetine (FLX)-treated mice were randomly assigned to VEH and 5-HT_2A_R antagonist (MDL) groups. Thirty minutes before behavioral testing, FLX mice received i.p. injections of either vehicle (0.5% DMSO) or MDL (0.01 mg/kg in 0.5% DMSO). (**b**) Bar plot of spontaneous alternation rates of SAL-treated mice that were administered VEH (black) and FLX-treated mice that were administered VEH (red) or MDL (blue) in the Y-maze. (**c**) Bar plot of the total number of entries into all arms of the Y-maze. (**d**) Representative heat map of results of the three-chamber social interaction test with a novel mouse and object. The occupancy rate was normalized to the region with peak occupancy in the arena. (**e**) Bar plot of the times spent sniffing the novel mouse and object in the three-chamber social interaction task. (**f**) Bar plot of the social preference index (time spent sniffing the mouse/total time spent sniffing the mouse and the object). The dotted line indicates an equal preference (50%) (**g**) Representative heat map of results from the three-chamber social interaction test with a familiar mouse and novel mouse. (H) Bar plot of the times spent sniffing the familiar mouse and a novel mouse in the three-chamber social interaction task. (**i**) Bar plot of the social novelty preference index (time spent sniffing the novel mouse/total time spent sniffing both mice). Data are presented as mean ± SEM. (**e**) and (**h**) Two-way repeated measures ANOVA. (**b**), (**c**), (**f**) and (**i**) One-way ANOVA. * *p* < 0.05, ** *p* < 0.01, *** *p* < 0.001

Upregulation of 5-HT_2A_R signaling in PV neurons suppressed spontaneous network firing in L5 PFC microcircuits, resulting in the poor performance of FLX-treated mice on working memory and social recognition tests. Therefore, we examined whether the selective suppression of 5-HT_2A_R signaling would enhance the performance of FLX-treated mice in the spontaneous alternation test and social novelty recognition tests (Fig. 4a). I.P. injection of a 5-HT_2A_R antagonist (MDL) did not influence the general behavior of wild-type animals, when they tested in the open-field test and elevated zero maze at a given dosage (data not shown), but the application of the same dosage of MDL sufficiently reversed 5-HT-mediated changes in the excitability of L5 FS interneurons from FLX-treated mice (Fig. 3g). Intriguingly, acute administration of MDL effectively reversed the poor performance of FLX-treated mice on the Y-maze spontaneous alternation task (Fig. 4b, c). Furthermore, the acute MDL treatment did not alter the social preference index in FLX-treated mice (Fig. 4d-f), but rescued deficits in the novel recognition task in FLX-treated mice (Fig. 4g-h). Taken together, our data revealed that the acute suppression of augmented 5-HT_2A_R signaling in FLX-treated mice could rescue their behavioral deficits in working memory and social recognition memory.

## Discussion

We showed that prenatally FLX-treated mice exhibited deficits in a working memory task and social novelty recognition paradigm via enhanced inhibitory synaptic activities in the L5 neurons of the mPFC resulting from enhanced 5-HT_2A_R signaling in FS PV neurons. More importantly, the acute inhibition of 5-HT_2A_R signaling in FLX-treated mice successfully reversed the observed behavioral deficits. Although 5-HT generally plays a critical role in mammalian neuronal development and behavior, the causal relationship between alterations in 5-HT homeostasis during pregnancy and adverse behavioral consequences in adulthood is poorly understood. Previously, several studies have attempted to address this question using both genetic deletion of SERT and SSRI administration in rodents. However, these studies suffered from inconsistent behavioral phenotypes, which were partially due to the use of different rodent strains and the type, dosage, and timing of administered SSRIs (see Additional file 1: Table S1). In the present study, we adopted a treatment scheme similar to that of Noorlander et al. This treatment mimicked SSRI exposure before the 3rd trimester in humans, in which doctors recommend that pregnant women abstain from (or reduce the dose of) SSRIs during late pregnancy [21]. In this paradigm, we consistently observed behavioral deficits in Y-maze spontaneous alternation tasks in prenatally FLX-treated mice without anxiety-related behaviors. More importantly, SSRI-treated mice exhibited normal sociability but impaired preference for social novelty in the three-chamber test (Fig. 1g-i), which is strikingly similar to the behaviors of mice lacking integrin β3, whose activities are linked to 5-HT transport and the pathophysiology of hyperserotonemia and autism [40, 41], as well as other mice lacking genes associated with autism [42–44].

In contrast to previous studies by Ansorge et al. [13] and Noorlander et al. [21] we did not find an anxiety-like effect in adult mice in our experiments. This could be due to two reasons: First, Ansorge et al. [13] employed postnatal treatment from P4-P21 and the difference in timeline of exposure to SSRI between the two protocols could have resulted in different developmental effects on different brain circuits. While the timing of our protocol was identical to that of Noorlander et al., [21], the difference in dosage of FLX that was used (ours: 0.6 mg/kg/day; Noorlander et al.: 0.8 mg/kg/day) could have different effect on the maturation and/or modification of emotional brain circuits. Although the dose difference between two protocols seemed to be small, Noorlander et al. [21] reported an increased mortality rate of offspring, which we did not observe in ours.

We recorded spontaneous synaptic activities induced by local inhibitory and excitatory networks in single neurons within L5, a major output neuronal layer within the mPFC. A significant increase in the frequency of sIPSCs was observed in the mPFC of FLX-treated mice compared with that in SAL-treated mice (Fig. 2g-i), but the frequency of sEPSCs recorded in the same neurons was decreased (Fig. 2a-c). Because both sIPSCs and sEPSCs were recorded in the same L5 pyramidal neurons, these data indicate that the ratio of excitatory to inhibitory drive onto those neurons is abnormally low in FLX-treated mice. As increase in sIPSC frequency is caused by activity-dependent changes such as excitability, we next blocked APs in a slice with TTX and recorded mIPSCs in L5 pyramidal neurons. Intriguingly, significant differences in both frequency and amplitude of mIPSCs were not observed between FLX- and SAL-treated mice, indicating that the increased frequency of inhibitory currents observed in L5 mPFC neurons was due to activity-dependent GABAergic release. Because these excitatory neurons received inhibitory synaptic inputs from PV-positive FS interneurons, we measured the intrinsic excitability of L5 FS interneurons and observed a significant increase in intrinsic excitability without marked changes in channel properties or input resistance (Additional file 1: Figure S3). We reasoned that the compensatory augmentation of specific 5-HT receptors could arise from prolonged exposure to 5-HT due to SSRI treatment and observed a concurrent increase in two 5-HT receptors, 5-HT_1A_R and _2A_R, using qPCR analysis. Because of the lack of suitable antibodies against 5-HT receptors for immunohistochemical analyses, we performed electrophysiological recordings and pharmacology to test the contribution of the increased abundance of specific 5-HT receptors in the PFC of FLX-treated mice. Surprisingly, increases in activity- and 5-HT-dependent changes in the excitability of FS interneurons were mediated by 5-HT_2A_Rs, but not 5-HT_1A_Rs (Fig. 3d-g and Additional file 1: Figure S5). Although we do not clearly understand why 5-HT_2A_R signaling or expression was enhanced specifically in FS interneurons, Athilingam et al. recently showed that 5-HT treatment in FS interneurons resulted in the suppression of an inward-rectifying potassium conductance, which eventually lead to increased excitability of these interneurons via 5-HT_2A_Rs [45], which might explain the excitability change in FS interneurons upon chronic SSRI treatment.

Changes in sIPSC frequency or altered inhibitory drive relative to excitatory drive have been observed in other animal models of neurodevelopmental disorders in which working memory deficits have also been reported [29, 46–48]. Our findings support the hypothesis that environmental changes induced by a single drug during pregnancy elicits an imbalance in the inhibitory/excitatory drive onto major output neurons in the L5 microcircuits within the PFC and subsequently alters animal behavior through non-genetic, compensatory upregulation of unique classes of 5-HT receptors in specific neuronal types. However, the potential mechanism of this compensatory 5-HT-receptor upregulation in FS interneurons needs to be addressed. Furthermore, studies to determine whether L2/3 neurons in the mPFC or hippocampal neurons exhibit comparable changes in the intrinsic excitability of neurons and excitatory/inhibitory (E/I) imbalance in the network will be interesting. Our data also support the findings from recent optogenetic studies showing that acute modulation of the excitability of FS PV-positive interneurons within the mPFC elicits changes in network oscillation and the performance of cognitive flexibility behavior in mice [49, 50]. Therefore, measurements of network oscillations in FLX-treated mice during working memory or social novelty task using in vivo multi-electrode recordings will be exciting.

The increased 5-HT_2A_R-mediated changes in the excitability of FS interneurons in FLX-treated mice prompted us to test whether the behavioral deficits of these mice were modulated by the application of a 5-HT_2A_R antagonist, such as MDL. The impairments of working memory and social novelty recognition were rescued by an in vivo treatment with MDL (Fig. 4 b-i). It is intriguingly, because a MDL treatment rescues attentional performance deficits in phencyclidine-treated [51] and NMDAR antagonist-treated [52] rats. Because patients with schizophrenia, who usually exhibit working memory deficits, have very high 5-HT_2A_R occupancy in the frontal cortex [53], the enhanced 5-HT_2A_R activity observed in our study may represent a general pathogenic mechanism of behavioral deficits in some mental disorders, and systemic administration of MDL might potentially restore synaptic and behavioral deficits in patients with disorders with similar etiologies.

Taken together, our data revealed that altered prenatal 5-HT homeostasis results in mPFC-dependent behavioral deficits, and systemic modulation of the augmented 5-HT_2A_R sufficiently rescues these behavioral deficits. Furthermore, our findings may potentially provide new opportunities for the pharmacological treatment of patients who have been prenatally exposed to psychotropic medications during the prenatal period.

## Materials and methods

### Animals

Animals were housed in a specific pathogen-free facility maintained below 22 °C and 55% humidity under a 12-h light-dark cycle (lights on at 0700 h) with food and water provided ad libitum [54]. Timed pregnancy was achieved by breeding wild-type C57BL6J (The Jackson Laboratory) male mice with female mice.

### Immunohistochemistry

Mice were perfused with PBS, followed by 4% (*w*/*v*) paraformaldehyde in PBS (pH 7.4). Brains were harvested and fixed with 4% paraformaldehyde overnight, transferred to 30% (w/v) sucrose in PBS, and then cryosectioned into 40 μm thick slices. For immunohistochemistry, brain slices were permeabilized with 0.2% Triton X-100 in PBS for 10 min and transferred to blocking buffer (5% donkey serum, 2% BSA and 0.2% Triton X-100 in PBS) for 1 h at room temperature. Next, the sections were incubated with primary antibody for parvalbumin (1:1000 diluted in blocking buffer, PV-235, Swant) overnight at 4 °C. The sections were subsequently incubated with appropriate secondary antibodies (1:500 diluted in blocking buffer, anti-streptavidin Alexa Fluor 488, anti-mouse Alexa Fluor 555; Invitrogen) for 2 h at room temperature. All sections were then stained with DAPI (1:5000 diluted with 0.2% Triton X-100 in PBS, Sigma-Aldrich) and mounted with Fluorsafe (Merck Millipore). Images were captured using an LSM 710 confocal microscope (Zeiss).

### Electrophysiology

L5 neurons were studied in acute coronal slices of the medial prefrontal cortex (mPFC) from male postnatal day 90–150 mice. After the mice were anesthetized by inhalation of 5% isoflurane, they were decapitated, and their brains were quickly removed and chilled in ice-cold, high-magnesium cutting solution containing the following components (in mM): 110 ChCl, 26 NaHCO_3_, 3.2 KCl, 0.5 CaCl_2_, 7 MgCl_2_, 1.25 NaH_2_PO_4_, 10 glucose, 2 sodium pyruvate, and 3 ascorbate [55]. The pH was adjusted to 7.4 by saturation with carbogen (95% O_2_ and 5% CO_2_), and the osmolality was approximately 300 mOsmol/L. The isolated brain was glued onto the stage of a vibrating blade Compresstome (VF-200, Precisionary), and 300 μm-thick slices were cut. The slices were incubated at 34 °C for 30 min in the same solution and thereafter maintained at room temperature. For experiments, we transferred a slice that recovered for at least one hour to a recording chamber superfused with artificial cerebrospinal fluid (aCSF) containing the following components (in mM): 124 NaCl, 26 NaHCO_3_, 3.2 KCl, 2.5 CaCl_2_, 1.3 MgCl_2_, 1.25 NaH_2_PO_4_, and 10 glucose. The aCSF was bubbled with 95% O_2_ and 5% CO_2_. Whole-cell voltage- or current-clamp recordings (one cell per slice) were performed at 32 ± 1 °C, and the rate of aCSF perfusion was maintained at 1–1.5 ml min^− 1^. Recordings were performed in somata with a Multiclamp 700B amplifier. Patch pipettes for current-clamp mode were filled with internal solutions containing the following components (in mM): 115 K-gluconate, 20 KCl, 10 Na_2_-phosphocreatine, 10 HEPES, 2 Mg-ATP, 0.3 NaGTP, and 0.1% biocytin. For voltage-clamp recordings, we used internal solutions containing the following components (in mM): 120 Cs-methane sulfonate, 10 CsCl, 10 TEA-Cl, 1 MgCl_2_, 10 HEPES, 0.1 EGTA, 0.4 Tris-GTP, 3 Mg-ATP, and 5 Na_2_-phosphocreatine. We recorded series resistance throughout the experiments and excluded neurons with series resistance > 20 MΩ from data analysis. Membrane potential values were presented as recorded without correcting for liquid junction potentials. MDL10090, CNQX, APV, picrotoxin, and TTX were purchased from Tocris Bioscience. All other drugs were purchased from Sigma-Aldrich. Stock solutions of drugs were made by dissolving in deionized water or DMSO according to manufacturer’s specifications and were stored at − 20 °C. On the day of the experiment, one aliquot was thawed and used. The concentration of DMSO in solutions was maintained at 0.1%.

### Behavioral assays and analyses

#### Animals and fluoxetine treatment

Male and female mice breeders were co-housed until pregnancy. The date when a plug was first noted was classified as ED0. From ED4 to ED19, pregnant females were housed individually and received daily i.p. injections of either FLX (0.6 mg/kg/day in a volume of 10 ml/kg, Sigma) or equal volumes of SAL [21]. Females used for injection were used only once to minimize the potential cross-generation effects of FLX administration. At postnatal day 21–23, offspring were weaned and housed with their same-sex littermates.

#### Behavioral assays

For behavioral testing, we used adult males treated prenatally with either FLX or SAL. All animals were 12 weeks old at the time of testing. All tests except for the open-field test were conducted during the light phase. All behavioral apparatuses were cleaned with 70% ethanol and dried with tissue between each animal.

#### Open-field test

The open-field test was conducted to measure general exploratory behavior. Mice were placed into the center of a Plexiglas cage (40.5 cm × 40.5 cm × 16 cm) for a 60-min test. Horizontal locomotion (i.e., distance traveled) was automatically recorded and analyzed by using Versamax software (AccuScan Instruments Inc).

#### Elevated zero maze

Anxiety-related behavior was measured using the elevated zero maze (CSI-MZ-ZR, Cleversys), which consists of a circular runway subdivided into two closed and two open sections elevated 60 cm above the floor. Mice were initially introduced into one of the closed runways and were allowed to freely explore the apparatus for 10 min. During the 10-min test, the percentage of time spent on the open arms and the number of transitions between two closed arms were scored using Topscan software (Cleversys).

#### Y-maze test

To allow the mice to discriminate between the three arms of the Y-maze apparatus (San Diego Instruments), the sides of one arm of was lined with a pattern of black vertical bars on a white background with a black square at the end. The sides of another arm were lined with a pattern of solid circles with black triangles on a white background and a triangle marking the end. The remaining arm was not marked. The Y-maze test was conducted for 10 min. The first minute was not coded and treated as the habituation period. Subsequently, the entries into each arm were recoded. The spontaneous alternation index was calculated as the number of non-repeating triplets (for example, if each arm was labeled “A”, “B” or “C”, “ABCAC” = 2) divided by the total number of possible non-repeating triplets (total number of entries into each arm - 2).

#### Novel object recognition test

The novel object recognition test was conducted in the open-field apparatus, which was subdivided equally into two arenas with a red plastic divider. For training, two identical objects constructed out of Lego blocks were placed at either end of the arena. Mice were allowed to explore and familiarize themselves with the two objects for 10 mins. They were returned to their cages for 20 min while the arena and the objects were cleaned to remove any odor cues. Afterwards, a test of short-term memory was conducted by placing the familiar object and a novel object that differed in shape, color, and size at either end of the arena. The duration of sniffing and bouts of sniffing were manually recorded using Topscan software (CleverSys).

#### Social cognition tests

Social preference and social recognition were tested using a three-chamber apparatus (CSI-SO-PP, CleverSys). The entire test consists of three consecutive 10 min blocks. For the first block, mice were habituated to the entire apparatus and two empty wire pencil holders placed at opposite corners. For the second block, one juvenile male mouse (social stimulus) was placed in one holder, and a dummy object (non-social stimulus) was placed in the other holder to test for social preference. For the last block, the juvenile mouse remained in one holder (familiar), but the dummy object was replaced by a novel juvenile male mouse (novel) to test for social discrimination.

### Golgi staining and tracing

Mice were perfused with PBS and placed in impregnation solution (FD Rapid Golgi-Stain Kit, FD Neurotech) for one week. 100 μm sections were cut on a cryostat, processed using the kit’s standard staining procedure and images were taken with the confocal microscope. Z-stack images were traced and analyzed with the Simple Neurite Tracer plugin for ImageJ.

### Gene expression study

Total RNA was extracted from punched samples of the mPFC using an RNeasy kit (Qiagen) followed by cDNA synthesis using a Quantitect Reverse Transcription kit (Qiagen). Real-time polymerase chain reaction (PCR) was performed on three independent sets of templates using iQ SYBR Green Supermix (Bio-Rad). The amplification mixture consisted of 1 μM primers, 10 μl of iQ SYBR Green Supermix (Bio-Rad), and 100 ng of template DNA in a total volume of 20 μl. Cycling parameters were 95 °C for 15 s, 57 °C for 1 min and 72 °C for 40 cycles using a CFX96 real-time PCR detection system (Bio-Rad). For each assay, PCR was performed after melting curve analysis. To reduce variability, we ran each sample in duplicate and included control qPCR reactions without the template in each run.

For microarray analyses, we normalized all microarray data using RMA method provided by the affy package [56] and analyzed differentially expressed genes using the limma package [57]. For gene-probe mapping information and GO term annotation, we used the EnsEMBL database (version 90).

### Statistical analyses

Data were analyzed and plotted using GraphPad Prism (GraphPad Software) and are presented as the means ± SEM with symbols representing individual subjects. Data from the open-field test data were analyzed using two-way repeated-measures ANOVA, followed by Tukey’s post hoc test. The rest of the data were analyzed using the Mann-Whitney test, Wilcoxon Signed rank test, and unpaired t-test. *P* < 0.05 was considered statistically significant. In addition, extreme values were subjected to Grubb’s test for outliers (http://graphpad.com/quickcalcs/Grubbs1.cfm) and excluded from the analysis.

## Additional file

Additional file 1: **Figure S1.** Prenatal FLX treatment does not induce other behavioral deficits. **Figure S2.** The prenatal FLX treatment does not change the morphology or spine density of L5 PrL neurons. **Figure S3.** Passive membrane properties of FS interneurons in the PrL of SAL- and FLX- treated mice. **Figure S4.** Expression of the mRNAs encoding serotonergic receptors and transporters and effects of the 5-HT treatment on IPSC frequency and amplitude. **Figure S5.** Effect of 5-HT1AR antagonists on FS interneurons in the PrL of SAL- and FLX-treated mice. **Figure S6.** Effects of the 5-HT treatment on pyramidal neurons in the PrL of SAL- and FLX-treated mice. **Table S1.** Summary of studies investigating the effects of perinatal serotonin reuptake inhibitors (SSRI) on adult male mice. **Table S2.** Intrinsic properties of fast-spiking interneurons of SAL and FLX treated mice before and after 5HT-treatment. **Table S3.** Statistical analysis conducted for each behavioral test. (PDF 1403 kb)

## Abbreviations

5-HT (serotonin): 5-hydrotryptamine
5-HT_2A_R: 5-HT 2A receptor
APV: (2R)-amino-5-phosphonopentanoic acid
FLX: Fluoxetine
FS: Fast-spiking
mEPSCs: Miniature excitatory postsynaptic currents
mIPSCs: Miniature inhibitory postsynaptic currents
mPFC: Medial prefrontal cortex
NMDA: *N*-methyl-*D*-aspartate
PrL: prelimbic area
PV: Parvalbumin
SAL: Saline
sEPSCs: Spontaneous excitatory postsynaptic currents
SERT: Serotonin transporter
sIPSCs: Spontaneous inhibitory postsynaptic currents
SSRI: Selective serotonin reuptake inhibitor
TTX: Tetrodotoxin
