# Supplementary material for: Prenatal selective serotonin reuptake inhibitor (SSRI) exposure induces working memory and social recognition deficits by disrupting inhibitory synaptic networks in male mice
Source: Mol Brain. 2019 Apr 1;12:29. doi: 10.1186/s13041-019-0452-5 (PMC6444596; doi:10.1186/s13041-019-0452-5)
Supplement: Supplementary file 1 — Figure S1. Prenatal FLX treatment does not induce other behavioral deficits. Figure S2. The prenatal FLX treatment does not change the morphology or spine density of L5 PrL neurons. Figure S3. Passive membrane properties of FS interneurons in the PrL of SAL- and FLX- treated mice. Figure S4. Expression of the mRNAs encoding serotonergic receptors and transporters and effects of the 5-HT treatment on IPSC frequency and amplitude. Figure S5. Effect of 5-HT1AR antagonists on FS interneurons in the PrL of SAL- and FLX-treated mice. Figure S6. Effects of the 5-HT treatment on pyramidal neurons in the PrL of SAL- and FLX-treated mice. Table S1. Summary of studies investigating the effects of perinatal serotonin reuptake inhibitors (SSRI) on adult male mice. Table S2. Intrinsic properties of fast-spiking interneurons of SAL and FLX treated mice before and after 5HT-treatment. Table S3. Statistical analysis conducted for each behavioral test. (PDF 1403 kb) [file 13041_2019_452_MOESM1_ESM.pdf]

Supplementary Figure Legends.

Supplementary Figure 1. Prenatal FLX treatment does not induce other behavioral deficits

(A) Bar plot of total distance travelled in the open field by SAL- (black) and FLX-treated (red) mice. **(B)** Bar plot of total time spent in the center of the open field. **(C)** Bar plot of time spent in the open arms of the elevated zero maze. **(D)** Bar plot of number of transitions between light and dark areas of the light-dark box test. **(E)** Bar plot of novel object preference in the novel object recognition task (time spent sniffing the novel object/total time spent sniffing both the familiar and novel objects) **(F)** Bar plot of total time spent grooming. **(G)** Bar plot of time spent sniffing left and right empty holders for SAL-treated mice in the social interaction chamber. **(H)** Bar plot of time spent sniffing left and right empty holders for FLX-treated mice in the social interaction chamber. Data are presented as mean ± SEM. All data were analyzed using the unpaired t-test. * $p < 0.05$

Supplementary Figure 2. The prenatal FLX treatment does not change the morphology or spine density of L5 PrL neurons

(A) Representative trace of Golgi-stained pyramidal neurons in L5 of the PrL. **(B)** Sholl analysis of dendritic intersections **(C)** Representative images of spine density on dendritic segments of pyramidal neurons in L5 of the PrL in SAL- and FLX- treated mice. **(D)** Bar plot of spine density. Data are presented as means ± SEM. **(B)** Two-way repeated measures ANOVA. **(D)** Unpaired t-test.

Supplementary Figure 3. Passive membrane properties of FS interneurons in the PrL of SAL- and FLX- treated mice

(A-D) Bar plots of **(A)** input resistance, **(B)** resting membrane potential, **(C)** AP threshold and **(D)** afterhyperpolarization amplitude recorded from the PrL of SAL- (black) and FLX-treated (red) treated mice. Data are presented as means ± SEM. All data were analyzed using the Wilcoxon signed ranks test.

Supplementary Figure 4. Expression of the mRNAs encoding serotonergic receptors and transporters and effects of the 5-HT treatment on IPSC frequency and amplitude.
(A-C) Bar plots of levels of **(A)** 5-HT-$_{1A}$R **(B)** 5-HT-$_{2A}$R and **(C)** Slc6a4 mRNAs, as assessed by qPT-PCR**. (D)** Gene chip analysis of the expression of 5-HT receptors and modulators **(E)** Representative current traces depicting sIPSCs obtained from pyramidal neurons in SAL- and FLX- treated mice. Bath application of 5-HT increased both the frequency and amplitude of sIPSCs in neurons from each group. **(F)** Bar plot summarizing the effects of 5-HT. **(A-C)** Unpaired t-test. **(F)** Data are presented as means ± SEM. Wilcoxon signed ranks test. * $p < 0.05$.

Supplementary Figure 5. Effect of 5-HT$_{1A}$R antagonists on FS interneurons in the PrL of SAL- and FLX-treated mice.
(A) Characteristic responses of FS interneurons from SAL-treated mice to current injections (200 pA) at baseline (CTRL), after the bath application of WAY-100135 (WAY) and after the bath application of 5-HT (5-HT). **(B)** Bar graphs summarizing the effects of WAY-100135 and 5-HT. **(C)** Characteristic responses of FS interneurons from FLX-treated mice to current injections (200 pA) under different condition. **(D)** Bar graphs summarizing the effects of WAY-100135 and 5-HT. Data are presented as means ± SEM. Wilcoxon signed ranks test. ** $p < 0.01$

Supplementary Figure 6. Effects of the 5-HT treatment on pyramidal neurons in the PrL of SAL- and FLX-treated mice.

(A) Representative traces showing that the bath application of 5-HT to spiking pyramidal neurons held at ~ -48 mV in current-clamp mode suppressed the firing frequency. **(B)** Characteristic responses of pyramidal neurons from SAL-treated mice (100 pA) before (Ctrl) and after the 5-HT bath application (5-HT). **(C)** Plot of the average mean number of spikes versus the current intensity obtained from pyramidal neurons of SAL-treated mice before and after 5-HT application. **(D)** Bar plot of sEPSC frequency obtained from pyramidal neurons of SAL-treated mice held at -70 mV. **(E)** Characteristic responses of pyramidal neurons from FLX-treated mice to current injections (100 pA) before (Ctrl) and after the 5-HT bath application (5-HT). **(F)** Plot of the average mean firing number of spikes versus the current intensity obtained from pyramidal neurons of FLX-treated mice before and after 5-HT application. **(G)** Bar plot of sEPSC frequency obtained from pyramidal neurons of FLX treated mice held at -70 mV. Data are presented as means ± SEM. Wilcoxon signed ranks test. ** $p < 0.01$.

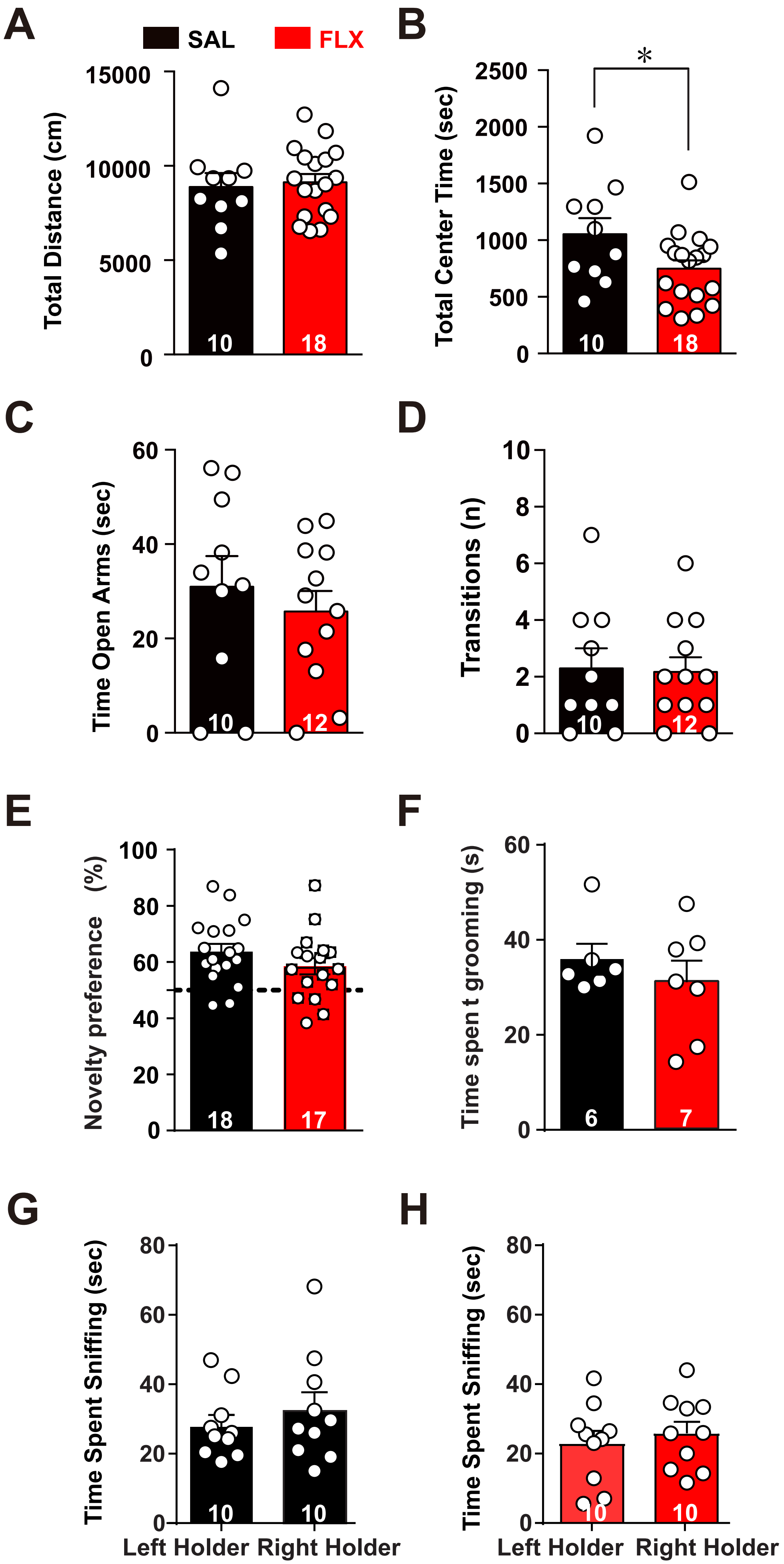
A
SAL
FLX
Total Distance (cm)
15000
10000
5000
0
10
18
B
Total Center Time (sec)
2500
2000
1500
1000
500
0
*
10
18
C
Time Open Arms (sec)
60
40
20
0
10
12
D
Transitions (n)
10
8
6
4
2
0
10
12
E
Novelty preference (%)
100
80
60
40
20
0
18
17
F
Time spen t grooming (s)
60
40
20
0
6
7
G
Time Spent Sniffing (sec)
80
60
40
20
0
10
10
Left Holder
Right Holder
H
Time Spent Sniffing (sec)
80
60
40
20
0
10
10
Left Holder
Right Holder

Figure S1

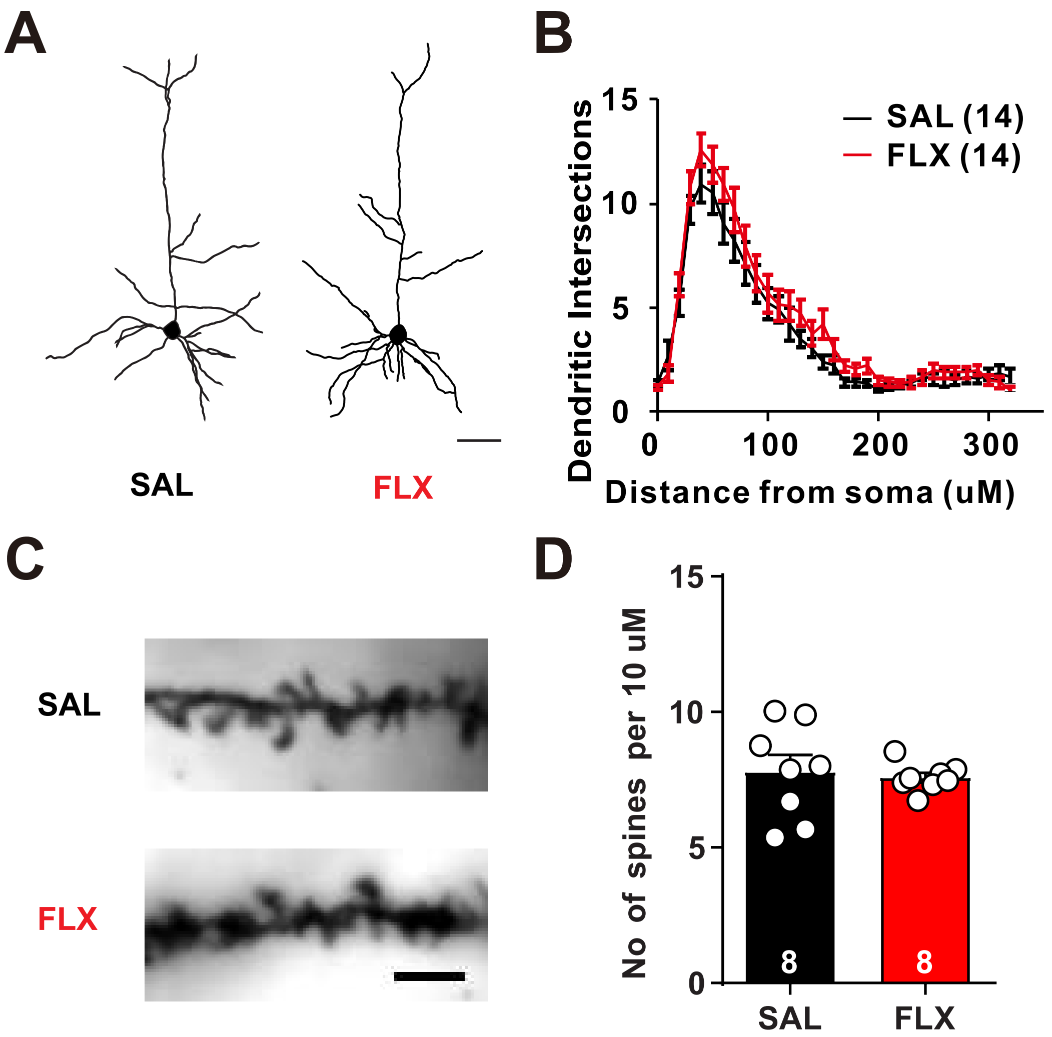
A
SAL
FLX
B
SAL (14)
FLX (14)
Dendritic Intersections
15
10
5
0
0
100
200
300
Distance from soma (uM)
C
SAL
FLX
D
No of spines per 10 uM
15
10
5
0
8
8
SAL
FLX

Figure S2

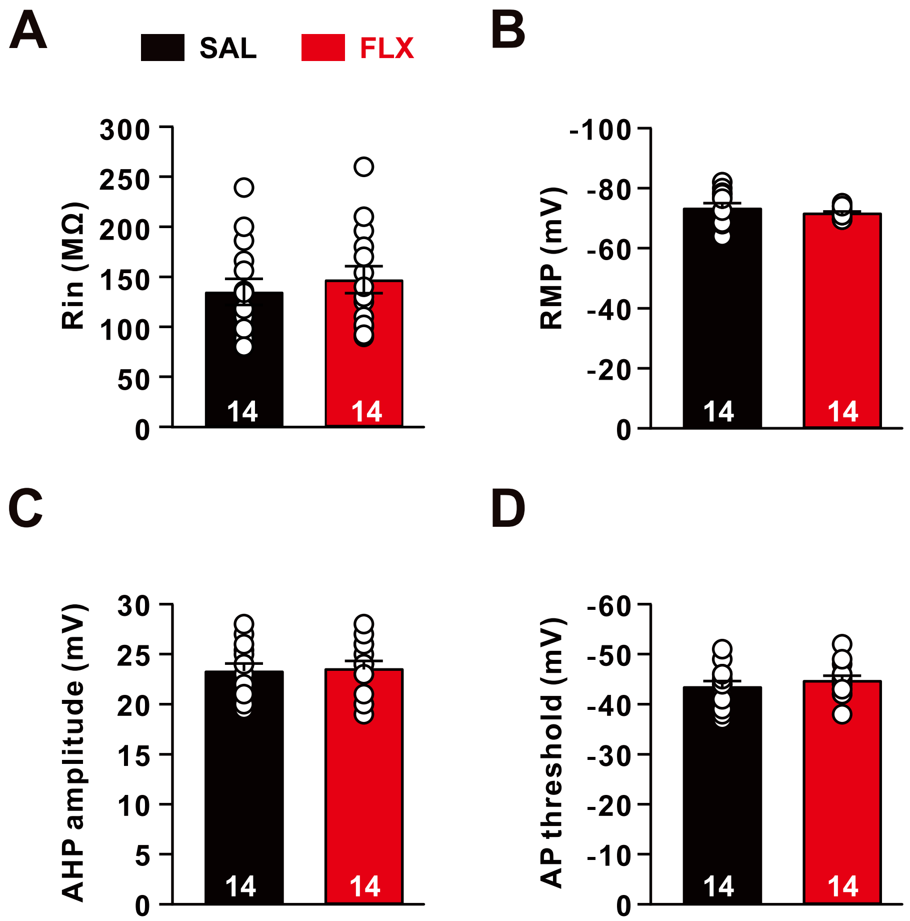

A
SAL
FLX
B
300
250
200
150
100
50
0
Rin (MΩ)
14
14
-100
-80
-60
-40
-20
0
RMP (mV)
14
14
C
D
30
25
20
15
10
5
0
AHP amplitude (mV)
14
14
-60
-50
-40
-30
-20
-10
0
AP threshold (mV)
14
14

Figure S3

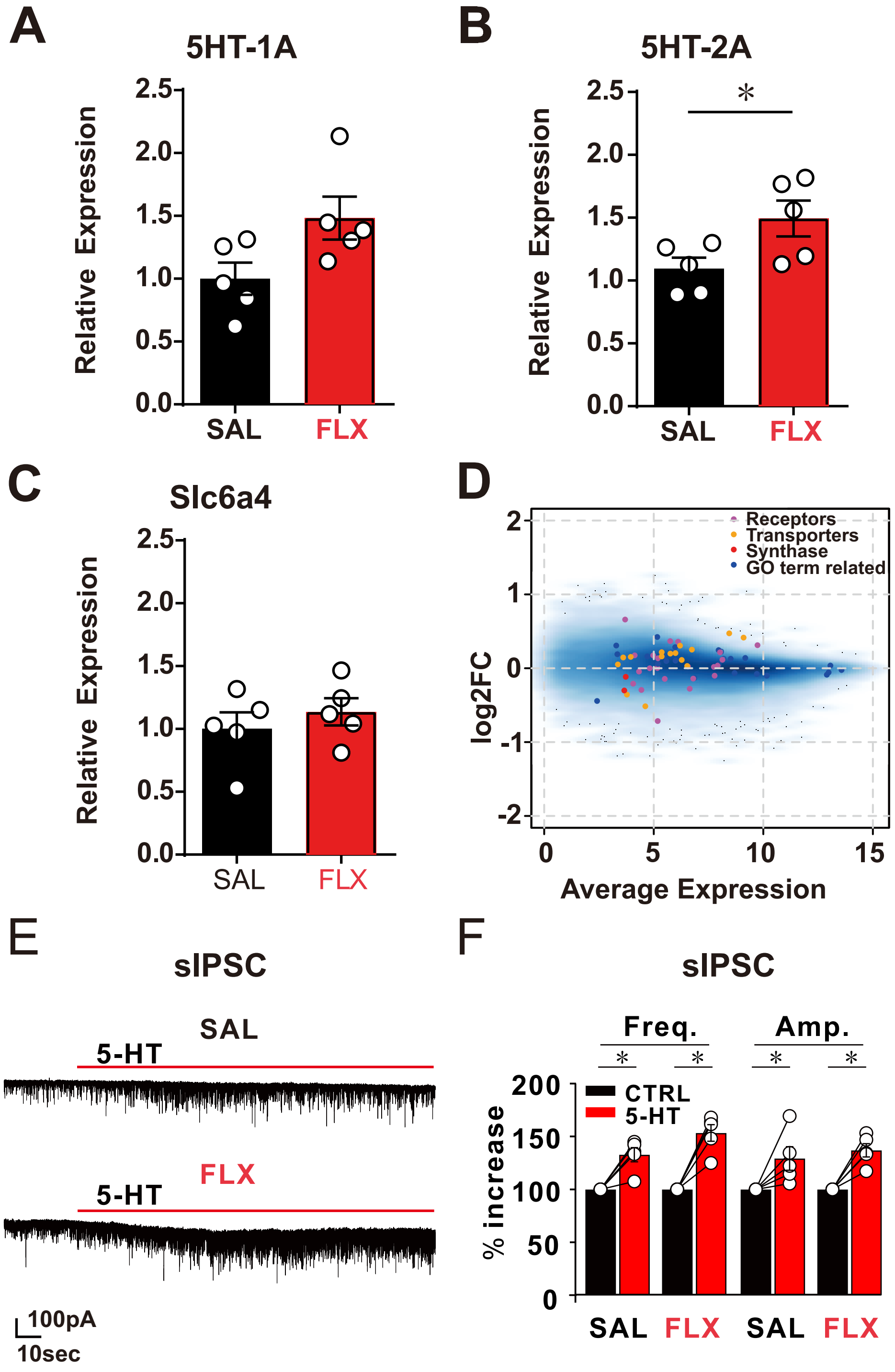
A
5HT-1A
Relative Expression
2.5
2.0
1.5
1.0
0.5
0.0
SAL
FLX
B
5HT-2A
*
Relative Expression
C
Slc6a4
Relative Expression
D
Receptors
Transporters
Synthase
GO term related
log2FC
Average Expression
E
sIPSC
SAL
5-HT
FLX
100pA
10sec
F
sIPSC
Freq.
Amp.
CTRL
5-HT
% increase
200
150
100
50
0

Figure S4

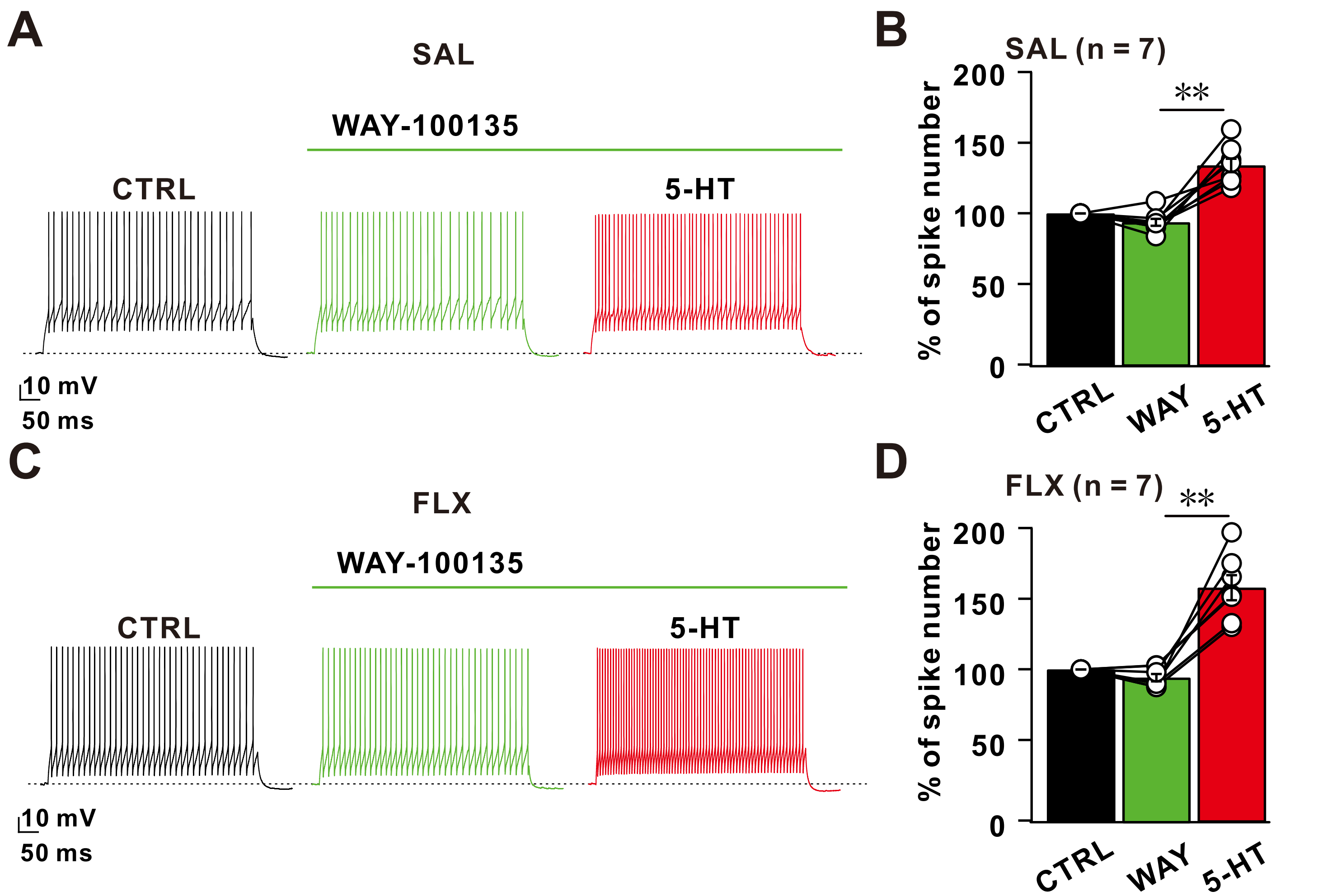
A
SAL
WAY-100135
CTRL
5-HT
10 mV
50 ms
B
SAL (n = 7)
% of spike number
200
150
100
50
0
**
CTRL
WAY
5-HT
C
FLX
WAY-100135
CTRL
5-HT
10 mV
50 ms
D
FLX (n = 7)
% of spike number
200
150
100
50
0
**
CTRL
WAY
5-HT

Figure S5

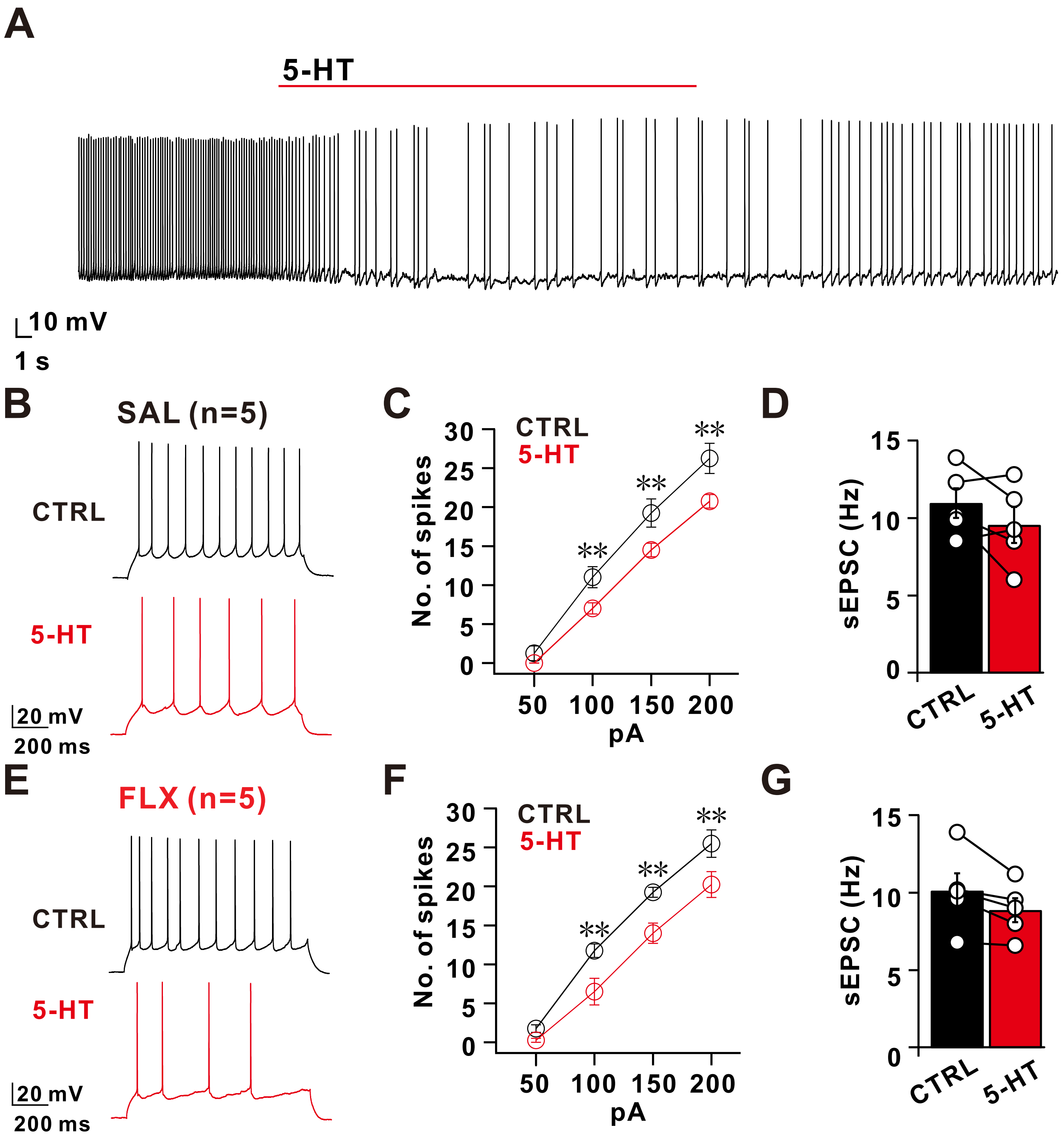

A
5-HT
10 mV
1 s
B
SAL (n=5)
CTRL
5-HT
20 mV
200 ms
C
CTRL
5-HT
No. of spikes
pA
D
sEPSC (Hz)
CTRL
5-HT
E
FLX (n=5)
CTRL
5-HT
20 mV
200 ms
F
CTRL
5-HT
No. of spikes
pA
G
sEPSC (Hz)
CTRL
5-HT

Figure S6

Supplementary Table 1. Summary of studies investigating the effects of perinatal serotonin reuptake inhibitors (SSRI) on adult male mice. Studies are arranged in order of window of SSRI exposure. EPM – Elevated Plus Maze, FST – Forced Swim Test, MWM – Morris Water Maze, NIN – Novelty Induced Hypophagia, NSF – Novelty Suppressed Feeding, OF – Open Field, RI – Resident Intruder, SA – Shock Avoidance, SE – Social Exploration

**Time of exposure**	**Drug and Dosage**	**Route of administration**	**Strain**	**Effects**	**Ref**
E0-P21	Fluoxetine (7.5mg/kg)	Gavage to dams daily	Swiss	↓ Locomotion in OF at P40 only	*(1)*
E0-P0	Fluoxetine (10mg/kg)	Intraperitoneal injections to dams daily	CD1	↑ Aggression in SE	*(2)*
E0-P14	Sertraline (5mg/kg for dams,1.5mg/kg for pups)	Intraperitoneal injections to dams daily during pregnancy<br><br>Intraperitoneal injections to pups daily	C57/BL6	No behavioural phenotype was observed	*(3)*
E8-E18	Fluoxetine (0.3, 0.6, 0.8 mg/kg)<br>Fluvoxamine (4.2mg/kg)	Intraperitoneal injections to dams daily	C57/BL6J	↑ Mortality (Dilated Cardiomyopathy) (Fluoxetine)<br>↑ Anxiety-like effect in EPM/OF/NSF (Fluoxetine/ Fluvoxamine)	*(4)*
E15-P12	Fluoxetine (25mg/kg)	Drinking water to dams	C57/BL6	↓ Anxiety-like effect in EPM<br>↑ Aggression in RI	*(5)*
E15-P12	Fluoxetine (25mg/kg)	Drinking water to dams	C57/BL6	↓ Anxiety-like effect in EPM<br>↑ Aggression in RI	*(6)*

				↑ Spatial memory in MWM	
P4-P21	Fluoxetine (10mg/kg)	Intraperitoneal injections to pups daily	129S6/SvEv	↓ Locomotor activity in OF<br>↑ Anxiety-like effect in EPM/OF<br>↑ Latency in SA	*(7)*
P4-P21	Fluoxetine (10mg/kg)<br>Citalopram (10mg/kg)<br>Clomipramine (20mg/kg)	Intraperitoneal injections to pups daily	129S6/SvEv	↓ Locomotor activity in OF<br>↑ Anxiety-like effect in EPM/OF/NSF/NIN<br>↑ Latency in SA	*(8)*
P4-P21	Fluoxetine (10 mg/kg)	Intraperitoneal injections to pups daily	C57/BL6J	↓ Body weight<br>↓ Locomotor activity in OF<br>↓ Depressive-like effect in FST	*(9)*
P5-P21	Fluoxetine (10mg/kg)<br>Escitalopram (10mg/kg)	Intraperitoneal injections to pups daily	CD-1/129SvEv	↓ Anxiety-like effect in EPM (Escitalopram)<br>↑ Anxiety-like effect in OF (Fluoxetine)	*(10)*

<u>**References**</u>

1. S. F. S. Lisboa, P. E. Oliveira, L. C. Costa, E. J. Venâncio, E. G. Moreira, Behavioral Evaluation of Male and Female Mice Pups Exposed to Fluoxetine during Pregnancy and Lactation, *Pharmacology* **80**, 49–56 (2007).

2. N. Svirsky, S. Levy, R. Avitsur, Prenatal exposure to selective serotonin reuptake inhibitors (SSRI) increases aggression and modulates maternal behavior in offspring mice: Prenatal SSRI Affects Social and Maternal Behavior, *Developmental Psychobiology* **58**, 71–82 (2016).

3. L. R. Meyer, B. Dexter, C. Lo, E. Kenkel, T. Hirai, R. D. Roghair, S. E. Haskell, Perinatal SSRI exposure permanently alters cerebral serotonin receptor mRNA in mice but does not impact adult behaviors, *The Journal of Maternal-Fetal & Neonatal Medicine* , 1–9 (2017).

4. C. W. Noorlander, F. F. T. Ververs, P. G. J. Nikkels, C. J. A. van Echteld, G. H. A. Visser, M. P. Smidt, D. N. Albertson, Ed. Modulation of Serotonin Transporter Function during Fetal Development Causes Dilated Heart Cardiomyopathy and Lifelong Behavioral Abnormalities, *PLoS ONE* **3**, e2782 (2008).

5. V. Kiryanova, S. J. Meunier, H. A. Vecchiarelli, M. N. Hill, R. H. Dyck, Effects of maternal stress and perinatal fluoxetine exposure on behavioral outcomes of adult male offspring, *Neuroscience* **320**, 281–296 (2016).

6. V. Kiryanova, R. H. Dyck, Increased Aggression, Improved Spatial Memory, and Reduced Anxiety-Like Behaviour in Adult Male Mice Exposed to Fluoxetine Early in Life, *Developmental Neuroscience* **36**, 396–408 (2014).

7. M. S. Ansorge, Early-Life Blockade of the 5-HT Transporter Alters Emotional Behavior in Adult Mice, *Science* **306**, 879–881 (2004).

8. M. S. Ansorge, E. Morelli, J. A. Gingrich, Inhibition of Serotonin But Not Norepinephrine Transport during Development Produces Delayed, Persistent Perturbations of Emotional Behaviors in Mice, *Journal of Neuroscience* **28**, 199–207 (2008).

9. N. N. Karpova, J. Lindholm, P. Pruunsild, T. Timmusk, E. Castrén, Long-lasting behavioural and molecular alterations induced by early postnatal fluoxetine exposure are restored by chronic fluoxetine treatment in adult mice, *European Neuropsychopharmacology* **19**, 97–108 (2009).

10. S. C. Altieri, H. Yang, H. J. O'brien, H. M. Redwine, D. Senturk, J. G. Hensler, A. M. Andrews, Perinatal vs genetic programming of serotonin states associated with anxiety, *Neuropsychopharmacology* **40**, 1456 (2015).

Supplementary Table 2. Intrinsic properties of fast-spiking interneurons of SAL and FLX treated mice before and after 5HT-treatment. RMP – resting membrane potential, R_{in} – input resistance, AP – action potential, AHP – afterhyperpolarization potential, mV – millivolt, MΩ – megaohm, ms – millisecond.

FSI	SAL	FLX	SAL(5HT)	FLX(5HT)
n	14	14	14	14
RMP (mV)	-73.43 ± 1.57	-70.14 ± 1.36	-71.83 ± 0.45	-67.36 ± 0.62
R_{in} (MΩ)	134.88 ± 13.03	147.07 ± 13.57	171.29 ± 13.51	187.86 ± 5.01
AP Halfwidth (ms)	0.21 ± 0.01	0.20 ± 0.01	0.22 ± 0.01	0.22 ± 0.01
AHP (mV)	23.35 ± 0.72	23.35 ± 0.74	23.57 ± 0.74	24.07 ± 0.44
AP Threshold (mV)	-43.57 ± 1.06	-43.36 ± 1.07	-44.79 ± 0.91	-44.71 ± 1.02

Supplementary Table 3. Statistical analysis conducted for each behavioral test.

Figure 1

**Test**	**Parameter**	**SAL**	**FLX**	**Statistical Method**	**t value or F value**	**p value**	**Multiple Comparisons**
Y-maze	Alternation Rate	n = 10	n = 18	Unpaired t-test	t = 3.142, df = 26	p = 0.0042 **	-
	Total Arm Entries	n = 10	n = 18	Unpaired t-test	t = 0.5384, df = 26	p = 0.5949	-
Crawley Box	Social Preference	n = 10	n = 10	Two-way ANOVA repeated measure	Main Effect of Group: F (1, 18) = 0.7003	p = 0.4137	SAL: object vs. mouse p < 0.001 *** FLX: object vs. mouse p < 0.001 ***
	Social Preference (%)	n = 10	n = 10	Unpaired t-test	t = 0.07839, df = 18	p = 0.9384	-
	Social Novelty	n = 10	n = 10	Two-way ANOVA repeated measure	Main Effect of Group: F (1, 18) = 3.935	p = 0.0628	SAL: familiar vs. stranger p = 0.0168 * FLX: familiar vs. stranger p = 0.9546
					Interaction: F (1, 18) = 5.222	p = 0.0347 *	
	Social Novelty (%)	n = 10	n = 10	Unpaired t-test	t = 0.2.623, df = 18	p = 0.0173 *	-

Figure 4

Test	Parameter	SAL (A)	FLX Veh (B)	FLX M100907 (C)	Statistical Method	t value or F value	p value	Multiple Comparisons
Y-maze	Alternation Rate	n = 10	n = 8	n = 9	One-way ANOVA	F (2, 24) = 4.472	p = 0.0224 *	(A) vs. (B) p = 0.1136<br>(A) vs. (C) p = 0.6182<br>(B) vs. (C) p = 0.0193 *
	Total Arm Entries	n = 10	n = 8	n = 9	One-way ANOVA	F (2, 24) = 2.654	p = 0.0909	(A) vs. (B) p = 0.6967<br>(A) vs. (C) p = 0.0768<br>(B) vs. (C) p = 0.3733
Crawley Box	Social Preference	n = 10	n = 10	n = 10	Two-way ANOVA repeated measure	Main Effect of Group: F (2, 27) = 3.445	p = 0.0465 *	(A) (B) (C)<br>object vs. mouse p < 0.001 ***
	Social Preference (%)	n = 10	n = 10	n = 10	One-way ANOVA	F (2, 27) = 1.307	p = 0.2873	(A) vs. (B) p = 0.2905<br>(A) vs. (C) p = 0.4608<br>(B) vs. (C) p = 0.9414
	Social Novelty	n = 10	n = 10	n = 10	Two-way ANOVA repeated measure	Main Effect of Group: F (2, 27) = 10.93<br>Interaction: F (2, 27) = 3.918	p = 0.0003 ***<br>p = 0.0321 *	(A) familiar vs. novel p = 0.0039 **<br>(B) familiar vs. novel p = 0.5974

								(C) familiar vs. novel p < 0.001 ***
	Social Novelty (%)	n = 10	n = 10	n = 10	One-way ANOVA	F (2, 27) = 4.157	p = 0.0267 *	(A) vs. (B) p = 0.0665<br>(A) vs. (C) p = 0.9575<br>(B) vs. (C) p = 0.0363 *

Figure S1

**Test**	**Parameter**	**SAL**	**FLX**	**Statistical Method**	**t value or F value**	**p value**	**Multiple Comparisons**
Open Field	Total Distance	n = 10	n = 18	Unpaired t-test	t = 0.3203, df = 26	p = 0.7513	-
	Total Center Time	n = 10	n = 18	Unpaired t-test	t = 2.121, df = 26	p = 0.0437 *	-
O-maze	Time Open Arm	n = 10	n = 12	Unpaired t-test	t = 0.6961, df = 20	p = 0.4944	-
	Transitions	n = 10	n = 12	Unpaired t-test	t = 0.1559, df = 20	p = 0.8777	-
Novel Object Recognition	Novelty preference	n = 18	n = 17	Unpaired t-test	t = 1.307, df = 33	p = 0.2003	-
Grooming	Time spent grooming	n = 6	n = 7	Unpaired t-test	t = 0.8394, df = 11	p = 0.4191	-

Figure S4

Experiment	Parameter	SAL	FLX	Statistical Method	t value or F value	p value	Multiple Comparisons
qPCR	5HT1A	n = 5	n = 5	Unpaired t-test	t = 2.248, df = 8	p = 0.0547	-
	5HT2A	n = 5	n = 5	Unpaired t-test	t = 2.389, df = 8	p = 0.0440 *	-
	Slc6a4	n = 5	n = 5	Unpaired t-test	t = 0.7991, df = 8	p = 0.4473	-
